# Identification of Risk Factors for Coexisting Sinusitis and Inflammatory Bowel Disease

**DOI:** 10.1093/crocol/otab054

**Published:** 2021-08-02

**Authors:** Victoria Rai, Cindy Traboulsi, Alexa Silfen, Max T Ackerman, Amarachi I Erondu, Jordan E Karpin, George Gulotta, David T Rubin

**Affiliations:** University of Chicago Medicine Inflammatory Bowel Disease Center, Chicago, Illinois, USA

**Keywords:** inflammatory bowel disease, sinusitis, comorbidities, multidisciplinary care, management

## Abstract

**Background:**

This study aimed to analyze the association of coexisting sinusitis and inflammatory bowel disease (IBD), establish significant factors involved in their development, and enable further biological correlation between these 2 diseases.

**Methods:**

The IBD and Sinusitis Study at UChicago Medicine (TISSUe) is a retrospective, single-center study. We reviewed patients to confirm IBD and chronic sinusitis diagnoses. Case-control propensity score matching was performed using matched controls with IBD only or sinusitis only. Statistical methods included chi-squared test and Wilcoxon rank sum test. Logistic regression analysis was performed, and factors were considered significant if *P* < .05.

**Results:**

Stratifying 214 patients with coexisting IBD and sinusitis, 176 patients had IBD first and 38 patients had sinusitis first. Multivariable analysis of factors associated with subsequent disease with matched controls determined that duration of disease, UC, steroid exposure ever, and younger age of IBD diagnosis were associated with subsequent sinusitis in patients with IBD; steroid exposure ever and duration of sinusitis were significantly associated with subsequent IBD in patients with sinusitis.

**Conclusion:**

This study suggests that IBD maintenance therapies are not associated with increased risk of sinusitis, as proposed by adverse events in clinical trial data; rather, UC diagnosis and duration of disease may be more influential in sinusitis development. While further studies are necessary, this study also demonstrates that sinusitis precedes IBD in some patients, probing its biological association with IBD and possible classification as an extraintestinal manifestation.

## Graphical Abstract

**Figure:**
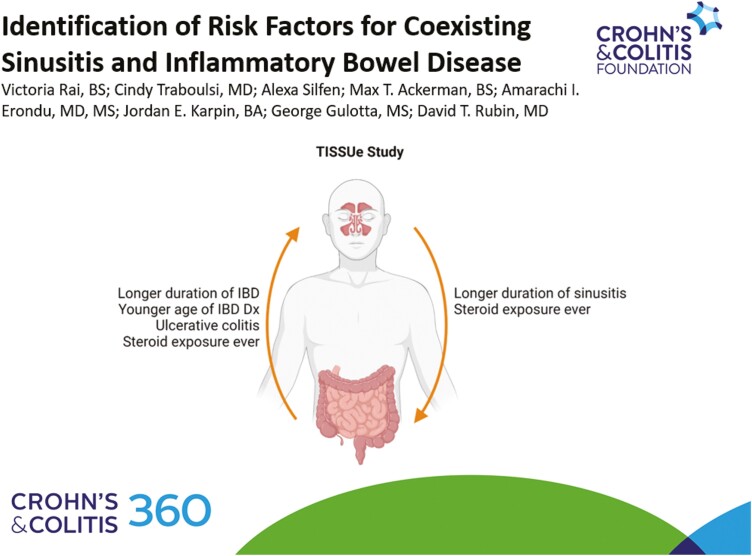

## Introduction

The association of sinusitis and inflammatory bowel disease (IBD) is not well known and neither are the risk factors for sinusitis development that may be specific to the IBD population. Prior studies did not identify premorbid IBD as a risk factor for chronic sinusitis.^1, 2^ One recent study identified IBD to be associated with 1- to 2-fold greater risk of subsequent chronic rhinosinusitis compared to the general population.^3^ However, these studies do not consider IBD-specific factors such as medication exposure, prolonged chronic inflammation, and bowel obstruction that have been found to pose additional risks to this population.^4^ Upper respiratory infections like sinusitis are commonly reported adverse events in clinical drug trials and real-world studies in patients with IBD.^5–8^ Though these studies suggest that sinusitis resulted from exposure to the drug of interest, there have been no studies linking drug exposures to subsequent sinusitis development in patients with IBD. With a lack of literature, it is highly relevant to establish factors associated with chronic sinusitis in the IBD population.

Sinusitis can be infectious or inflammatory in origin, as well as acute or chronic. Chronic sinusitis is described as inflammation of the sinus or nasal passages for more than 12 weeks per episode and usually requires therapeutic or surgical intervention to resolve. Patients having more than 4 episodes of sinusitis in 1 year would be considered to have recurrent sinusitis, while acute sinusitis is more short-term and triggered by cold or allergies. Sinusitis may occur with or without nasal polyps, and its etiology may be rooted in infectious or inflammatory origins. Medical management of chronic sinusitis may include nasal sprays, steroids, antihistamines, decongestants, antibiotics, or surgery. Though the etiology and pathophysiology of sinusitis is multifactorial, it is thought that chronic sinusitis and IBD share innate immune and epithelial barrier dysfunction of the nasal and gut mucosa respectively.^2, 9, 10^ This shared immune dysfunction, combined with evidence from trials and anecdotal patient experiences, warrant exploration of sinusitis, particularly sinusitis that is chronic and persistent in nature, in patients with IBD.

Medical management of IBD focuses on the achievement of disease remission and prevention of disease flares, but also includes management of extraintestinal manifestations (EIM) and comorbidities that co-occur with IBD.^11^ Many EIMs are inflammatory in origin, much like sinusitis, and can impact the musculoskeletal, ocular, and dermatologic systems such as arthritis, uveitis, and psoriasis. Though a majority of EIMs are subsequently diagnosed, some may precede IBD diagnosis.^12^ Though chronic sinusitis is not classified as an EIM, its seeming commonality among the IBD population from clinical trial data and real-world studies and its similar presentation to other EIMs is curious and warrants investigation. Upper airway and nasopharyngeal diseases may be lesser known but commonly occurring conditions in IBD populations and co-maintenance of these conditions are vital for patient quality of life.

This study aimed to analyze the association of coexisting sinusitis and IBD, establish risk factors involved in the development of sinusitis in patients with IBD, consider the development of IBD in patients with sinusitis, and enable further biological correlation between these 2 diseases.

## Methods

### Data Collection

The IBD and Sinusitis Study at UChicago Medicine (TISSUe) is a retrospective study at our tertiary IBD center. We utilized our institution’s electronic medical record data warehouse of 2.4 million patients to identify patients age ≥18 years with diagnostic codes for chronic sinusitis (ICD-9: 473, ICD-10: J32) and IBD (ICD-9: 555/556, ICD-10: K50/K51) between January 1999 and May 2019. Diagnostic codes on record indicate ongoing conditions that the patient is seen for at our institution, designated before or during referral for such condition. This search identified 14 753 patients with only sinusitis, 13 980 patients with only IBD, and 386 patients with both sinusitis and IBD. A random 1% subset of patients with only sinusitis and with only IBD underwent manual chart review in order to confirm a diagnosis of IBD or sinusitis in the patient chart to ensure accuracy and reliability of this large dataset.

We collected demographic and clinical information including sex, disease type, age at disease diagnosis, and smoking history. We included bowel obstruction using ICD codes (ICD-9: 560.9, ICD-10: K56.60 and K56.69) in our data collection. Medication data pertaining to ever using 5-aminosalicylates (5-ASAs), immunomodulators, anti-TNFs, anti-integrins, anti-IL12/23s, JAK inhibitors, or steroids were also collected for each patient. Age of diagnosis of disease was of interest and is presented by clinical category. For IBD, the Montreal classification grouped patients with an IBD diagnosis at ≤16 years old into A1, 17–40 years old into A2, and over 40 years old into A3.^13^ For sinusitis, clinical consensus from an expert panel identified that management of chronic sinusitis in pediatric patients ≤12 years old is distinctly different from management of pediatric patients 13–18 years old who may be more similar to adults.^14^ Therefore, this analysis characterized the sinusitis age classification as pediatric ≤12, pediatric 13–18, and adult being over 18 years old.

Duration of disease was gathered from date of disease diagnosis to date of data retrieval. Date of first sinonasal symptoms was recorded as well as date of a formal diagnosis of sinusitis. Due to uncertainty of chronicity upon first sinonasal symptoms delaying time of diagnosis, we used age of first sinonasal symptoms in our analyses of age of disease onset.

### Patient Inclusion

**Figure 1.**
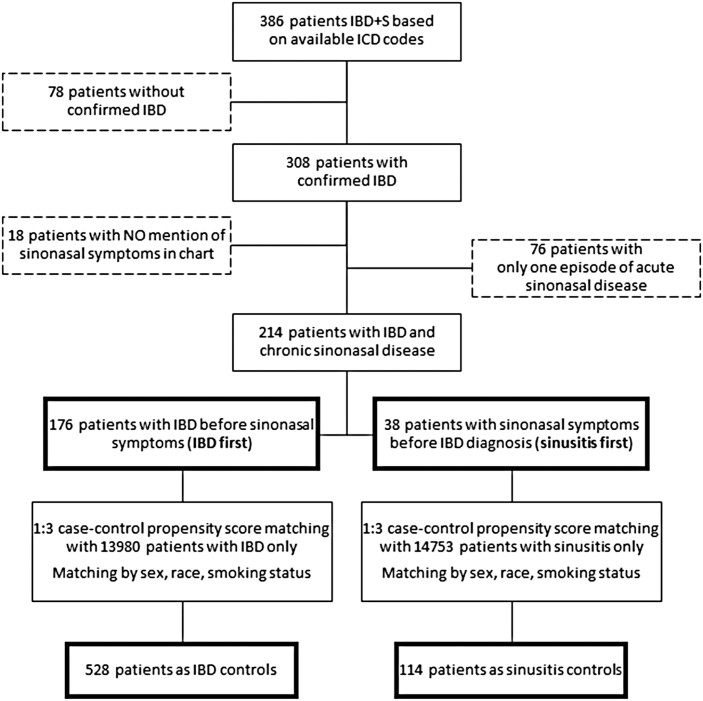
Inclusion criteria for patients in IBD + S cohort based on chart review and case-control propensity matching criteria for controls in TISSUe database.

From the original TISSUe database, 386 patients had IBD and sinusitis (IBD + S) based on available ICD codes. Manual chart review was done to ensure that all of the patients included in this study were confirmed to have each disease by objective definitions. These patients were reviewed to confirm chronic IBD diagnosis (ulcerative colitis [UC], Crohn’s disease [CD], or indeterminate colitis) on endoscopic and histologic findings. Chronic sinusitis was confirmed by 2 criteria: (1) two or more symptoms of drainage, congestion, facial pain/pressure, or decreased sense of smell experiences for 12 weeks or longer and (2) documentation of objective evidence on physical exam or imaging indicating inflammation, cloudy mucous, polyps in nasal cavity, or edema.^15^ Ultimately, 78 patients were excluded for not having confirmed chronic IBD and 94 patients were excluded for not having chronic, recurrent, or ongoing sinonasal disease meeting these criteria (Figure 1).

### Power Calculation

Without preconceived knowledge of the effect sizes, we estimated needing 197 patients to achieve 80% power for conventionally small, medium, and large effect sizes using methodology described by Cohen et al.^16^ To compare patients with IBD + S to respective controls, we estimated needing between 24 and 393 patients in each group to achieve 80% power for detecting conventionally small, medium, and large effect sizes.

### Matching Criteria

Case-control propensity score matching was conducted on R version 3.6.3 using the MatchIt package and nearest neighbor methodology. Using the TISSUe database to match controls, a 1:3 case-control matching was performed with 2 groups: first between patients with IBD then sinusitis (IBD first) and IBD only, second between patients with sinusitis then IBD (sinusitis first) and sinusitis only. Patients were matched for sex, race, and smoking status which were considered strong confounders as risk factors for both IBD and sinusitis (Figure 1).^17–22^ These groups were used to predict factors associated with subsequent disease development in the respective group on univariable and multivariable logistic regression analysis.

### Statistical Analysis

Statistical analyses were performed on R version 3.6.3 (R Foundation for Statistical Computing, Vienna, Austria). Parametric variables were analyzed using chi-squared test, and non-parametric variables were analyzed using Wilcoxon rank sum test.

After case-control propensity score matching, logistic regression analysis assessed the factors associated with the disease outcome of interest, either development of sinusitis in patients with IBD or development of IBD in patients with sinusitis. Factors significant at *P* < .10 on univariable analysis were included in subsequent multivariable analysis. Factors were considered significant if *P* < .05. Adjusted odds ratios (aOR) were reported with 95% confidence intervals (95% CI).

### Ethical Considerations

This study was approved by the University of Chicago’s Institutional Review Board (IRB19-0107).

## Results

### Characteristics of Patients With Coexisting Sinusitis and IBD (IBD + S)

The 20-year prevalence of sinusitis in the IBD population at our institution was 1.5%, significantly greater than the 20-year prevalence of sinusitis in the total institutional population of 0.61% (*P* < .0001).

In the 214 patients with confirmed IBD + S, most were of female sex (65.4%), white (72.4%), never smokers (81.8% and had CD (57%). In terms of sinusitis history, 57.9% were seen by an ENT regarding their sinusitis, 79% had ever been exposed to antibiotics for their sinonasal disease and nearly 30% of patients had a sinus surgery. The average age of IBD diagnosis was 29.9 years old, the average age of first recorded sinonasal symptoms was 38.9 years old, while the average age of sinusitis diagnosis was 40.6 years old. The average duration of IBD was 20.7 years, and the average duration of sinusitis was 11.7 years. Regarding IBD medication exposure ever, 44.4% of patients had been on 5-ASAs, 29% on immunomodulators, 36.4% on anti-TNFs, 7% on anti-integrins, 5.6% on anti-interleukins, and 0.9% on JAK inhibitors. While steroid exposure may be due to either IBD or sinusitis, 77.6% of patients had ever been exposed to steroids (Table 1).

**Table 1.** Demographics of IBD + S cohort and comparison of characteristics stratified by diagnosis of IBD first (IBD → S) and diagnosis of sinusitis first (S → IBD)

	IBD + S, *N* = 214	IBD → S	S → IBD
Of total IBD + S, *n* (%)		176 (82.2%)	38 (17.8%)
Female sex, *n* (%)	140 (65.4%)	116 (65.9%)	24 (63.2%)
Race, *n* (%)			
White	155 (72.4%)	129 (73.3%)	26 (68.4%)
Black/African-American	52 (24.3%)	41 (23.3%)	11 (28.9%)
Asian	3 (1.4%)	2 (1.1%)	1 (2.6%)
Other	4 (1.9%)	4 (2.3%)	0 (0.0%)
Ever smoker, *n* (%)	39 (18.2%)	33 (18.8%)	6 (15.8%)
IBD type, *n* (%)			
Crohn’s disease	122 (57.0%)	103 (58.5%)	19 (50.0%)
Ulcerative colitis	87 (40.7%)	71 (40.4%)	16 (42.1%)
Indeterminate colitis	5 (2.3%)	2 (1.1%)	3 (7.9%)
Bowel obstruction, *n* (%)	35 (16.4%)	32 (18.2%)	3 (7.9%)
Seen by ENT, *n* (%)	124 (57.9%)	99 (56.3%)	25 (65.8%)
Sinus surgery, *n* (%)	64 (29.9%)	54 (30.7%)	10 (26.3%)
Average time to first surgery from first sinonasal symptoms, years (SD)	4.3 (9.2)	3.5 (7.4)	8.4 (15.8)
Antibiotic exposure for sinonasal disease ever, *n* (%)	169 (79.0%)	141 (80.1%)	28 (73.7%)
IBD medication exposure ever, *n* (%)			
5-ASAs	95 (44.4%)	74 (42.0%)	21 (55.3%)
IMMs	62 (29.0%)	52 (29.5%)	10 (26.3%)
Anti-TNFs	78 (36.4%)	71 (40.4%)	7 (18.4%)
Anti-integrins	15 (7.0%)	14 (7.95%)	1 (2.6%)
Anti-interleukins	12 (5.6%)	9 (5.1%)	3 (7.9%)
JAK inhibitors	2 (0.9%)	2 (1.1%)	0 (0.0%)
Steroids exposure ever, *n* (%)	166 (77.6%)	137 (77.8%)	29 (76.3%)
Average age at IBD diagnosis, years (SD)	29.9 (15.1)	28.5 (14.3)	36.5 (17.2)
Average age of first sinonasal symptoms, years (SD)	38.9 (16.9)	40.8 (16.4)	29.9 (16.5)
Average time between first sinonasal symptoms and IBD diagnosis, years (SD)	11.3 (10.7)	12.3 (10.8)	6.7 (8.9)
Average age at sinusitis diagnosis, years (SD)	40.6 (16.8)	41.7 (16.4)	35.2 (17.8)
Average duration of IBD, years (SD)	20.7 (12.5)	22.8 (12.2)	10.7 (8.4)
Average duration of sinusitis, years (SD)	11.7 (8.1)	10.5 (5.8)	17.4 (13.2)
Montreal classification, *n* (%)			
A1 (≤16 years old)	42 (19.6%)	37 (21.0%)	5 (13.2%)
A2 (17–40 years old)	123 (57.5%)	104 (59.1%)	19 (50.0%)
A3 (>40 years old)	49 (22.9%)	35 (19.9%)	14 (36.8%)
Sinusitis age classification, *n* (%)			
Pediatric ≤12	10 (4.7%)	4 (2.3%)	6 (15.8%)
Pediatric 13–18	15 (7.0%)	10 (5.7%)	5 (13.2%)
Adult	189 (88.3%)	162 (92.0%)	27 (71.0%)

Abbreviations: 5-ASAs, 5-aminosalicylates; IBD, inflammatory bowel disease; JAK, janus kinase inhibitors; IMMs, immunomodulators; S, sinusitis; TNFs, tumor necrosis factors.

The majority of patients with IBD + S were diagnosed with both diseases as an adult (*n* = 165, 77.1%) vs both diagnoses at a pediatric (A1 for IBD or pediatric sinusitis age classification) age (*n* = 18, 8.4%). The remaining patients were diagnosed at pediatric age for only IBD (*n* = 24, 11.2%) or only sinusitis (*n* = 7, 3.3%). There were significantly more patients with CD with adult sinusitis (*P* = .031) and with pediatric sinusitis age 13–18 (*P* = .012) than patients with UC (Supplementary Table).

Stratifying patients by order of disease diagnosis, 176 (82.2%) patients had IBD first and 38 (17.8%) patients had sinusitis first. Most characteristics were similar between these 2 groups (Table 1).

### Factors Associated With Subsequent Sinusitis in Matched IBD Controls

The 176 patients with IBD first were matched with patients with only IBD for sex, race, and smoking status (Table 2). Factors associated with sinusitis development in patients with IBD were identified on univariable analysis and included in multivariable analysis if they were significant. On multivariable analysis, steroid exposure ever (aOR 2.87, 95% CI, 1.90–4.40) and duration of IBD (aOR 1.12, 95% CI, 1.10–1.15) were significantly associated with increased odds of subsequent sinusitis diagnosis in patients with IBD. CD (aOR 0.39, 95% CI, 0.24–0.62) and IBD diagnosis in Montreal classification A3 (aOR 0.46, 95% CI, 0.24–0.91) were significantly associated with decreased odds of subsequent sinusitis in patients with IBD (Table 2). Bowel obstruction was associated with increased odds on univariable analysis (aOR 2.44, 95% CI, 1.49–3.99) but not on multivariable analysis. No IBD medication was significantly associated with subsequent sinusitis development in patients with IBD.

**Table 2.** Logistic regression analysis evaluating factors involved with sinusitis development in patients with IBD

	IBD only, *n* = 528	IBD → S, *n* = 176	Univariable OR (95% CI)	*P*	Multivariable aOR (95% CI)	*P*
Female sex, *n* (%)	348 (65.9%)	116 (65.9%)	Matched			
Race, *n* (%)			Matched			
White	387 (73.3%)	129 (73.3%)				
Black/African-American	123 (23.3%)	41 (23.3%)				
Asian	6 (1.1%)	2 (1.1%)				
Other	12 (2.3%)	4 (2.3%)				
Ever smoker, *n* (%)	99 (18.8%)	33 (18.8%)	Matched			
Crohn’s disease, *n* (%)	355 (67.2%)	103 (58.5%)	**0.67 (0.47–0.95)**	.026*	0.39 (0.24–0.62)	<.0001*
Bowel obstruction, *n* (%)	44 (8.3%)	32 (18.2%)	**2.44 (1.49–3.99)**	.0003*	**1.40 (0.75–2.57)**	.280
IBD medication exposure ever, *n* (%)						
5-ASAs	195 (36.9%)	74 (42.0%)	1.24 (0.87–1.75)	.227		
IMMs	129 (24.4%)	52 (29.5%)	1.30 (0.88–1.89)	.18		
Anti-TNFs	174 (32.9%)	71 (40.4%)	1.38 (0.97–1.95)	.075	1.14 (0.73–1.78)	.572
Anti-integrins	50 (9.5%)	14 (7.95%)	0.83 (0.43–1.49)	.545		
Anti-interleukins	24 (4.5%)	9 (5.1%)	1.13 (0.49–2.40)	.758		
JAK inhibitors	9 (1.7%)	2 (1.1%)	0.66 (0.10–2.60)	.601		
Steroids exposure ever, *n* (%)	280 (53.0%)	137 (77.8%)	**3.11 (2.12–4.67)**	<.0001*	**3.98 (2.46–6.62)**	<.0001*
Average age at IBD diagnosis, years (SD)	37.6 (18.4)	28.5 (14.3)	**0.968 (0.957–0.978)**	<.0001*		
Montreal classification, *n* (%)						
A1 (≤16 years old)	57 (10.8%)	37 (21.0%)	REF			
A2 (17–40 years old)	263 (49.8%)	104 (59.1%)	**0.61 (0.38–0.98)**	.039*	0.70 (0.41–1.26)	.228
A3 (>40 years old)	208 (39.4%)	35 (19.9%)	**0.26 (0.15–0.45)**	<.0001*	**0.46 (0.24–0.91)**	.025*
Duration of IBD, years (SD)	10.5 (8.4)	22.8 (12.2)	**1.12 (1.10–1.14)**	<.0001*	**1.12 (1.10–1.15)**	<.0001*

Values in bold are significant at *P* < .05.Abbreviations: 5-ASAs, 5-aminosalicylates; aOR, adjusted odds ratio; CI, confidence interval; IBD, inflammatory bowel disease; IMMs, immunomodulators; OR, odds ratio; TNFs, tumor necrosis factors.

*Significant at *P* < .05.

### Factors Associated With Subsequent IBD in Matched Sinusitis Controls

The 38 patients with sinusitis first were matched with patients with sinusitis only for sex, race, and smoking status (Table 3). Compared to patients with sinusitis who did not develop subsequent IBD, multivariable analysis identified duration of sinusitis (aOR 1.26, 95% CI, 1.14–1.41) and steroid exposure ever (aOR 6.17, 95% CI, 2.36–18.39) to be significantly associated with increased odds of subsequent IBD (Table 3). Pediatric age ≤12 years old (aOR 1.62, 95% CI, 0.36–6.48) and pediatric age 13–18 years old (aOR 2.11, 95% CI, 0.27–13.00) were associated with greater odds of developing IBD, though not significant on multivariable analysis.

**Table 3.** Logistic regression analysis evaluating factors involved with IBD development in patients with sinusitis

	Sinusitis only, *n* = 114	S → IBD, *n* = 38	Univariable OR (95% CI)	*P*	Multivariable aOR (95% CI)	*P*
Female sex, *n* (%)	72 (63.2%)	24 (63.2%)	Matched			
Race, *n* (%)			Matched			
White	78 (68.4%)	26 (68.4%)				
Black/African-American	33 (28.9%)	11 (28.9%)				
Asian	3 (2.6%)	1 (2.6%)				
Other	–	–				
Ever smoker, *n* (%)	18 (15.8%)	6 (15.8%)	Matched			
Bowel obstruction, *n* (%)	3 (2.6%)	3 (7.9%)	3.17 (0.57–17.82)	.169		
IBD medication exposure ever, *n* (%)						
5-ASAs	0 (0.0%)	21 (55.3%)				
IMMs	4 (3.5%)	10 (26.3%)				
Anti-TNFs	0 (0.0%)	7 (18.4%)				
Anti-integrins	0 (0.0%)	1 (2.6%)				
Anti-interleukins	0 (0.0%)	3 (7.9%)				
JAK inhibitors	0 (0.0%)	0 (0.0%)				
Steroids exposure ever, *n* (%)	47 (41.2%)	29 (76.3%)	**4.59 (2.06–11.12)**	.0003*	**6.17 (2.36–18.39)**	.0004*
Average age of first sinonasal symptoms, years (SD)	42.2 (20.8)	29.9 (16.5)	**0.97 (0.95–0.99)**	.002*		
Sinusitis age classification, *n* (%)						
Pediatric ≤12	11 (9.6%)	6 (15.8%)	1.98 (0.63–5.71)	.216	1.62 (0.36–6.48)	.507
Pediatric 13–18	5 (4.4%)	5 (13.2%)	2.63 (0.95–13.96)	.054	2.11 (0.27–13.00)	.431
Adult	98 (86.0%)	27 (71.0%)	REF			
Duration of sinusitis, years (SD)	8.9 (3.9)	17.4 (13.2)	**1.21 (1.12–1.33)**	<.0001*	**1.26 (1.14–1.41)**	<.0001*

Abbreviations: 5-ASAs, 5-aminosalicylates; aOR, adjusted odds ratio; CI, confidence interval; IBD, inflammatory bowel disease; IMMs, immunomodulators; OR, odds ratio; TNFs, tumor necrosis factors.

*Significant at *P* < .05.

## Discussion

This is the first study to consider the co-development of IBD and sinusitis, to establish IBD-specific risk factors associated with chronic sinusitis development, and to explore the association between sinusitis preceding IBD.

This study identified that most patients with coexisting IBD + S at our tertiary care center develop sinusitis an average of 12 years after their IBD diagnosis. We then sought to establish factors associated with the development of sinusitis in an IBD population. We compared patients with IBD who do and do not develop sinusitis. On the matched analysis, we demonstrated that steroid exposure ever, having UC, younger age of IBD diagnosis, and longer duration of IBD is associated with greater odds of sinusitis development. In addition, bowel obstruction was found significant on univariable analysis, yet its loss of significance on multivariable analysis may be attributed to accounting for duration of disease. A cross-sectional study in patients with IBD and chronic sinusitis found bowel obstruction to be associated with double the odds of sinusitis development, and attributed this relationship to altered motility patterns directly impacting the upper gastrointestinal system and, in turn, the upper airway.^4^ Notably, this analysis cannot determine if bowel obstruction that precedes or follows sinusitis diagnosis is associated with different odds. Patients diagnosed with IBD at age 16 or younger (Montreal classification A1) were associated with significantly greater odds of developing sinusitis which complements the finding that longer duration of IBD is also associated with sinusitis development. This relationship with age and duration could be explained by the immune-mediating nature of IBD, a longer burden of the disease from being diagnosed earlier may have immunologic consequences elsewhere. Further, alongside the finding that patients with UC have greater odds of developing sinusitis, our findings support the hypothesized relationship between the gut and nasal microbiome.^23, 24^ One study suggests that the physical process of swallowing may recolonize bacteria in the gut with bacteria of the sinus in patients with UC which may contribute to disease onset and progression.^23^

Our study does not find any IBD-specific immunosuppressive medications or biologics to be associated with subsequent sinusitis development. This finding does not preclude medication exposure as a trigger of sinusitis in patients with IBD but does suggest that biological underpinnings of sinusitis development in IBD may be more influential in onset. As such, the infectious or inflammatory classification of sinusitis is of interest to determine appropriate management strategies for sinusitis in the setting of IBD as underlying immunoglobulin deficiency may be implicated in both IBD and sinusitis.^10, 25, 26^

In addition to evaluating the risk of developing sinusitis in a matched IBD population, our analysis recognized that nearly 20% of patients with coexisting IBD + S had sinusitis and developed IBD. Matched analysis in the sinusitis population identified steroid exposure ever to be associated with 4.5-fold greater odds of subsequent IBD and pediatric age of sinusitis onset to be associated with about 2.6-fold greater odds. While no firm conclusions can be made by this analysis due to important limitations, these findings do suggest a closely linked association with earlier age and time to disease onset which are novel and have not yet been characterized. This relationship may be partly due to lead-time bias patients who have earlier and more frequent interaction with the medical system due to their earlier age of diagnosis with chronic diseases may be more likely to be diagnosed with another chronic disease. Overall, these findings continue to support the hypothesized anatomical and mucosal relationship between the gut and nasal microbiomes.^23, 24^

Of great interest is the role of antibiotic exposure in patients with sinonasal disease and eventual development of IBD. In the general population, antibiotic exposure has been highly associated with newly-onset IBD and particularly more common in pediatric CD.^27, 28^ Of the 8 patients who had sinusitis first and subsequent CD in this study, 6 (75%) patients had been exposed to antibiotics for sinusitis prior to diagnosis with CD. Studies have suggested that patients with antibiotic exposure due to their sinonasal disease, even only in the short term, may have an altered microbiome that could be causal to their IBD diagnosis.^29, 30^ The ability to determine whether an infection, such as a sinus infection, or antibiotic usage for said infection is associated with subsequent IBD is difficult; further studies are encouraged to explore these nuances to better understand the pathophysiology and etiology of chronic sinusitis in context to IBD.

This analysis was unable to assess the temporal nature of medication exposure with disease course and severity due to its retrospective nature. Given this limitation, this study is unable to draw greater conclusions regarding an association with medications, such as with steroids or antibiotics. This association may further be confounded by previous failure of other IBD medications due to prolonged or severe disease. Future studies must longitudinally assess disease activity, disease course, and medication administration in relation to both IBD and chronic sinusitis to account for the contributions of medication factors in disease risk. Another limitation lying in the retrospective nature of this study is the use of diagnostic codes to identify patients with IBD and/or sinusitis. Though patients were identified for the study based on diagnostic code, confirmation by manual chart review for objective definitions of IBD and sinusitis was necessary to provide a more robust inclusion criterion. Though a subset of the matching cohort was also chart reviewed to confirm diagnoses, future studies would benefit from prospective identification of a study and matching cohort based on these objective inclusion criteria. Other limitations of this analysis include limited data availability on other risk factors associated with IBD development and sinusitis development that were limited based on the retrospective nature of this study. Known risk factors for IBD include, and are not limited to, asthma, family history of IBD, history of infection, environmental and sociodemographic factors that are risk factors or early life predictors of IBD.^31, 32^ Known risk factors for sinusitis include structural problems with the sinuses (eg, nasal polyps, deviated septum), asthma and allergies, atopy, environmental quality, and occupational factors.^33^ Some of these conditions are thought to be co-occurring with IBD, so it can be difficult to discern the relationship between these diseases in addition to sinusitis without more temporal information that was limited by the retrospective nature of this study.^34^ Lastly, as a single tertiary center experience, multicenter and prospective studies are necessary to further confirm these findings in other populations.

This study aimed to understand the association of sinusitis and IBD that could enable further biological correlation, provide insight into the prevalence of sinusitis in IBD, and explore sinusitis as a possible EIM. It appears that sinusitis presentation can be preceding and subsequent to IBD, as is similar with other EIMs.^12^ Though this study alone cannot determine if sinusitis is an EIM of IBD, it adds to the body of literature that upper respiratory manifestations of IBD are of increasing interest.^35, 36^ One review article outlines how these manifestations can impact treatment options and clinical practice and supports that co-management of these diseases in patients with IBD is essential.^37^ This relationship is highly relevant, clinically and biologically, as shared mechanism of disease can lead to shared therapeutic pathways for treating these diseases.

It is undeniable that sinusitis affects a small proportion of the IBD population at our center over the last 20 years, our overall 20-year prevalence being about 1.5%. Though small, this is still significantly greater than the 20-year prevalence of sinusitis in the general population at our institution. In addition, nearly 60% of the 214 patients with IBD + S experienced significant enough symptoms to seek out care from an ENT specialist, almost 30% have had surgery related to their chronic sinusitis, about a quarter of whom underwent multiple surgeries. Even so, this study only captures patients with a chronic sinusitis diagnosis in their electronic medical record and not patients who may be suffering from long-standing sinus issues, potentially undiagnosed chronic sinusitis, that might have been unaddressed.^38^ We argue that, while small, there is an interesting association between sinusitis and IBD onset that should be given greater consideration and involved multidisciplinary care in order to improve patients’ quality of life. In patients with IBD and ongoing or recurring sinonasal symptoms, referral to ENT may be considered for evaluation of chronic sinonasal disease. Like IBD, early identification of sinonasal disease is key to prevent more serious disease and complications, both in sinusitis and IBD outcomes, downstream. Though our analysis found otherwise, this may be the case for any patient with IBD on therapies with highly reported sinonasal events in clinical trials and real-world experiences until further explanation of this association is identified.

## Conclusion

In summary, this study elucidates the importance of age of diagnosis and duration in disease onset between chronic sinonasal disease and IBD, rather than therapeutic interventions. Further studies are necessary to correlate the biological underpinnings of IBD and chronic sinonasal disease and confirm predictive factors of subsequent disease development.

## Supplementary Material

otab054_suppl_Supplementary_Table

## Data Availability

The data that support the findings of this study are available upon request from the corresponding author.

## References

[1] Tan BK , Chandra RK, Pollak J, et al. Incidence and associated pre-morbid diagnoses of patients with chronic rhinosinusitis. J Allergy Clin Immunol. 2013;131(5):1350–1360. doi:10.1016/j.jaci.2013.02.002 23541327 PMC3788631

[2] Chandra RK , Lin D, Tan B, et al. Chronic rhinosinusitis in the setting of other chronic inflammatory diseases. Am J Otolaryngol. 2011;32(5):388–391. doi:10.1016/j.amjoto.2010.07.013 20832903 PMC3387732

[3] Lin Y-H , Lin CL, Kao C-H. Adults with inflammatory bowel disease are at a greater risk of developing chronic rhinosinusitis: a nationwide population-based study. Clin Otolaryngol. 2021;46(1):196–205. doi:10.1111/coa.13647 32886858

[4] Book DT , Smith TL, McNamar JP, Saeian K, Binion DG, Toohill RJ. Chronic sinonasal disease in patients with inflammatory bowel disease. Am J Rhinol. 2003;17(2):87–90. doi:10.1177/194589240301700204 12751702

[5] Hamzaoglu H , Cooper J, Alsahli M, Falchuk KR, Peppercorn MA, Farrell RJ. Safety of infliximab in Crohn’s disease: a large single-center experience. Inflamm Bowel Dis. 2010;16(12):2109–2116. doi:10.1002/ibd.21290 20848473

[6] Cross RK , Chiorean M, Vekeman F, et al. Assessment of the real-world safety profile of vedolizumab using the United States Food and Drug Administration adverse event reporting system. PLoS One. 2019;14(12):e0225572. doi:10.1371/journal.pone.0225572 31800627 PMC6892509

[7] Christensen B , Colman RJ, Micic D, et al. Vedolizumab as induction and maintenance for inflammatory bowel disease: 12-month effectiveness and safety. Inflamm Bowel Dis. 2018;24(4):849–860. doi:10.1093/ibd/izx067 29562271 PMC6196763

[8] Weisshof R , Golan MA, Sossenheimer PH, et al. Real world experience with tofacitinib in IBD at a tertiary center. Dig Dis Sci. 2019;64(7):1945–1951. doi:10.1007/s10620-019-05492-y 30734234 PMC6935176

[9] Hoggard M , Mackenzie BW, Jain R, Taylor MW, Biswas K, Douglas RG. Chronic rhinosinusitis and the evolving understanding of microbial ecology in chronic inflammatory mucosal disease. Clin Microbiol Rev. 2017;30(1):321–348. doi:10.1128/CMR.00060-16 27903594 PMC5217796

[10] Bachert C , Marple B, Schlosser RJ, et al. Adult chronic rhinosinusitis. Nat Rev Dis Primer. 2020;6(1):1–19. doi:10.1038/s41572-020-00218-1 33122665

[11] Chams S , Badran R, Sayegh SE, Chams N, Shams A, Hajj Hussein I. Inflammatory bowel disease: looking beyond the tract. Int J Immunopathol Pharmacol. 2019;33:1–18. doi:10.1177/2058738419866567 PMC6685113 31382828

[12] Vavricka SR , Schoepfer A, Scharl M, Lakatos PL, Navarini A, Rogler G. Extraintestinal manifestations of inflammatory bowel disease. Inflamm Bowel Dis. 2015;21(8):1982–1992. doi:10.1097/MIB.0000000000000392 26154136 PMC4511685

[13] Spekhorst LM , Visschedijk MC, Alberts R, et al. Performance of the Montreal classification for inflammatory bowel diseases. World J Gastroenterol. 2014;20(41):15374–15381. doi:10.3748/wjg.v20.i41.15374 25386087 PMC4223272

[14] Brietzke SE , Shin JJ, Choi S, et al. Clinical consensus statement: pediatric chronic rhinosinusitis. Otolaryngol Neck Surg. 2014;151(4):542–553. doi:10.1177/0194599814549302 25274375

[15] Rosenfeld RM , Piccirillo JF, Chandrasekhar SS, et al. Clinical practice guideline (update): adult sinusitis. Otolaryngol Neck Surg. 2015;152(2_suppl):S1–S39. doi:10.1177/0194599815572097 25832968

[16] Cohen J. Statistical Power Analysis for the Behavioral Sciences. 2nd ed. Hillsdale, NJ: Lawrence Erlbaum Associates; 1988.

[17] Shah SC , Khalili H, Gower-Rousseau C, et al. Sex-based differences in incidence of inflammatory bowel diseases—pooled analysis of population-based studies from western countries. Gastroenterology. 2018;155(4):1079–1089.e3. doi:10.1053/j.gastro.2018.06.043 29958857

[18] Aniwan S , Harmsen WS, Tremaine WJ, Edward V, Loftus J. Incidence of inflammatory bowel disease by race and ethnicity in a population-based inception cohort from 1970 through 2010. Ther Adv Gastroenterol. 2019;12:1–8. doi:10.1177/1756284819827692 PMC6376543 30792818

[19] Parkes GC , Whelan K, Lindsay JO. Smoking in inflammatory bowel disease: impact on disease course and insights into the aetiology of its effect. J Crohns Colitis. 2014;8(8):717–725. doi:10.1016/j.crohns.2014.02.002 24636140

[20] Ference EH , Tan BK, Hulse KE, et al. Commentary on gender differences in prevalence, treatment, and quality of life of patients with chronic rhinosinusitis. Allergy Rhinol. 2015;6(2):e82–e88. doi:10.2500/ar.2015.6.0120 PMC4541639 26302727

[21] Soler ZM , Mace JC, Litvack JR, Smith TL. Chronic rhinosinusitis, race, and ethnicity. Am J Rhinol Allergy. 2012;26(2):110–116. doi:10.2500/ajra.2012.26.3741 22487286 PMC3345896

[22] Reh DD , Higgins TS, Smith TL. Impact of tobacco smoke on chronic rhinosinusitis – a review of the literature. Int Forum Allergy Rhinol. 2012;2(5):362–369. doi:10.1002/alr.21054 22696460 PMC3443524

[23] Yang P-C , Liu T, Wang B-Q, et al. Rhinosinusitis derived Staphylococcal enterotoxin B possibly associates with pathogenesis of ulcerative colitis. BMC Gastroenterol. 2005;5:28. doi:10.1186/1471-230X-5-28 16144553 PMC1215483

[24] Marsland BJ , Trompette A, Gollwitzer ES. The gut–lung axis in respiratory disease. Ann Am Thorac Soc. 2015;12(Supplement 2):S150–S156. doi:10.1513/AnnalsATS.201503-133AW 26595731

[25] Lee S , Lane AP. Chronic rhinosinusitis as a multifactorial inflammatory disorder. Curr Infect Dis Rep. 2011;13(2):159–168. doi:10.1007/s11908-011-0166-z 21365379 PMC4372071

[26] Schwitzguébel AJ-P , Jandus P, Lacroix J-S, Seebach JD, Harr T. Immunoglobulin deficiency in patients with chronic rhinosinusitis: systematic review of the literature and meta-analysis. J Allergy Clin Immunol. 2015;136(6):1523–1531. doi:10.1016/j.jaci.2015.07.016 26329513

[27] Ungaro R , Bernstein CN, Gearry R, et al. Antibiotics associated with increased risk of new-onset Crohn’s disease but not ulcerative colitis: a meta-analysis. Am J Gastroenterol. 2014;109(11):1728–1738. doi:10.1038/ajg.2014.246 25223575

[28] Kronman MP , Zaoutis TE, Haynes K, Feng R, Coffin SE. Antibiotic exposure and IBD development among children: a population-based cohort study. Pediatrics. 2012;130(4):e794–e803. doi:10.1542/peds.2011-3886 23008454 PMC4074626

[29] Theochari NA , Stefanopoulos A, Mylonas KS, Economopoulos KP. Antibiotics exposure and risk of inflammatory bowel disease: a systematic review. Scand J Gastroenterol. 2018;53(1):1–7. doi:10.1080/00365521.2017.1386711 29022402

[30] Jakobsson HE , Jernberg C, Andersson AF, Sjölund-Karlsson M, Jansson JK, Engstrand L. Short-term antibiotic treatment has differing long-term impacts on the human throat and gut microbiome. PLoS One. 2010;5(3):e9836. doi:10.1371/journal.pone.0009836 20352091 PMC2844414

[31] Kuenzig ME , Barnabe C, Seow CH, et al. Asthma is associated with subsequent development of inflammatory bowel disease: a population-based case–control study. Clin Gastroenterol Hepatol. 2017;15(9):1405–1412.e3. doi:10.1016/j.cgh.2017.02.042 28344063

[32] Bernstein CN , Burchill C, Targownik LE, Singh H, Roos LL. Events within the first year of life, but not the neonatal period, affect risk for later development of inflammatory bowel diseases. Gastroenterology. 2019;156(8):2190–2197.e10. doi:10.1053/j.gastro.2019.02.004 30772341 PMC7094443

[33] Min J-Y , Tan BK. Risk factors for chronic rhinosinusitis. Curr Opin Allergy Clin Immunol. 2015;15(1):1–13. doi:10.1097/ACI.0000000000000128 25479315 PMC4368058

[34] Kuenzig ME , Bishay K, Leigh R, Kaplan GG, Benchimol EI, Crowdscreen SR Review Team. Co-occurrence of asthma and the inflammatory bowel diseases: a systematic review and meta-analysis. Clin Transl Gastroenterol. 2018;9(9):188. doi:10.1038/s41424-018-0054-z 30250122 PMC6155154

[35] Eloy P , Leruth E, Goffart Y, et al. Sinonasal involvement as a rare extraintestinal manifestation of Crohn’s disease. Eur Arch Otorhinolaryngol. 2007;264(1):103–108. doi:10.1007/s00405-006-0146-3 17021783

[36] Baili L , Rachdi I, Daoud F, et al. A rare manifestation of Crohn’s disease: sinonasal granulomatosis. Report of a case and review of literature. Eur J Case Rep Intern Med. 2014;1:1–5. doi:10.12890/2014_000123

[37] Zois CD , Katsanos KH, Tsianos EV. Ear-nose-throat manifestations in inflammatory bowel diseases. Ann Gastroenterol. 2007;20(4):265–274.

[38] Lim CGT , Spanger M. Incidental maxillary sinus findings in patients referred for head and neck CT angiography. Singapore Dent J. 2012;33(1):1–4. doi:10.1016/j.sdj.2012.10.001 23739316

