# Supplementary material for: Identification of Risk Factors for Coexisting Sinusitis and Inflammatory Bowel Disease
Source: Crohns Colitis 360. 2021 Aug 2;3(3):otab054. doi: 10.1093/crocol/otab054 (PMC9075692; doi:10.1093/crocol/otab054)
Supplement: otab054_suppl_Supplementary_Table [file otab054_suppl_supplementary_table.docx]

Supplemental Table: Number of patients in IBD+S by disease by disease type, Montreal classification, and age of sinusitis symptoms

| **Montreal Classification** | **A1** |  | **A1 Total** | **A2** |  | **A2 Total** | **A3** |  | **A3 Total** |  |  | **Total in each Sinusitis Age Classification (n)** |
| --- | --- | --- | --- | --- | --- | --- | --- | --- | --- | --- | --- | --- |
| **Sinusitis Age Classification** | **UC** | **CD** |  | **UC** | **CD** |  | **UC** | **CD** |  | **Total UC** | **Total CD** |  |
| Adult | 5 | 19 | 24 | 48 | 69 | 117 | 31 | 17 | 48 | 84 | 105 | **189** |
| Pediatric 13-18 | 3 | 7 | 10 |  | 4 | 4 | 1 |  | 1 | 4 | 11 | **15** |
| Pediatric ≤12 | 3 | 5 | 8 | 1 | 1 | 2 |  |  |  | 4 | 6 | **10** |
|  |  |  |  |  |  |  |  |  |  |  |  |  |
| **Total in each Montreal Classification (n)** | **11** | **31** | **42** | **49** | **74** | **123** | **32** | **17** | **49** | **92** | **122** | **214** |
